# Supplementary material for: Characterization of Triadica sebifera (L.) Small Extracts, Antifeedant Activities of Extracts, Fractions, Seed Oil and Isolated Compounds against Plutella xylostella (L.) and Their Effect on Detoxification Enzymes
Source: Molecules. 2022 Sep 22;27(19):6239. doi: 10.3390/molecules27196239 (PMC9573571; doi:10.3390/molecules27196239)
Supplement: Supplementary file 1 [file molecules-27-06239-s001.zip › molecules-1853927-supplementary.pdf]

*Supplementary Materials*

# Characterization of *Triadica sebifera* (L.) Small Extracts, Antifeedant Activities of Extracts, Fractions, Seed Oil and Isolated Compounds against *Plutella xylostella* (L.) and Their Effect on Detoxification Enzymes

**Shudh Kirti Dolma [1,2] and S. G. Eswara Reddy [1,2,*]**

[1] Entomology Laboratory, Agrotechnology Division, CSIR-Institute of Himalayan Bioresource Technology, Palampur 176061, India
[2] Academy of Scientific and Innovative Research (AcSIR), Ghaziabad 201002, India
* Correspondence: ereddy@ihbt.res.in or ereddy2001@yahoo.com or sgereddy73@gmail.com

**Contents**

**Figure S1**. Feeding deterrence index of leaf and bark ethanol aqueous extract and seed oil of *Triadica sebifera* against *Plutella xylostella* with choice and without choice; The same letters in the error bars (Mean± SE) within the figure are not statistically different by Tukey's HSD ($P \leq 0.05$)

**Figure S2**. Feeding deterrence index of binary mixtures of seed oil with leaf and bark ethanol aqueous extract of *Triadica sebifera* against *Plutella xylostella* with choice and without choice; The same letters in the error bars (Mean± SE) within the figure are not statistically different by Tukey's HSD ($P \leq 0.05$)

**Figure S3.** Feeding deterrence index of leaf fractions of *Triadica sebifera* against *Plutella xylostella* with choice and without choice; The same letters in the error bars (Mean± SE) within the figure are not statistically different by Tukey's HSD ($P \leq 0.05$)

**Figure S4.** Feeding deterrence index of bark fractions of *Triadica sebifera* against *Plutella xylostella* with choice and without choice; The same letters in the error bars (Mean± SE) within the figure are not statistically different by Tukey's HSD ($P \leq 0.05$)

**Tables S1.** Growth inhibition of leaf/bark extracts, fractions and seed oil of *Triadica sebifera* against *Plutella xylostella*

**Tables S2.** Growth inhibition of, leaf, bark ethanol aqueous extracts, its fractions and seed oil of *Triadica sebifera* against *Plutella xylostella* (without choice method)

**Table S3.** Growth inhibition of binary mixtures of *Triadica sebifera* against *Plutella xylostella* (without choice)

**Figure S5.** Per cent repellence of leaf and bark fractions of *Triadica sebifera* against *Plutella xylostella*; The same letters in the error bars (Mean± SE) within the figure are not statistically different by Tukey's HSD ($P \leq 0.05$)

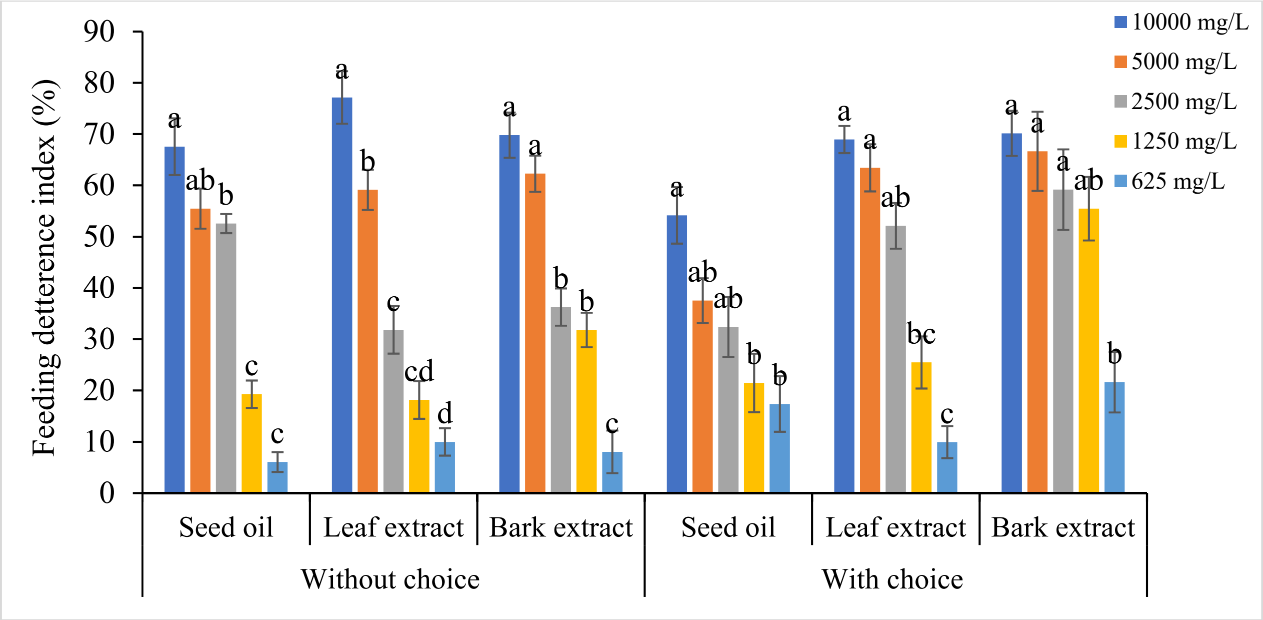


**Figure S1**. Feeding deterrence index of leaf and bark ethanol aqueous extract and seed oil of *Triadica sebifera* against *Plutella xylostella* with choice and without choice; The same letters in the error bars (Mean ± SE) within the figure are not statistically different by Tukey's HSD ($P \leq 0.05$)

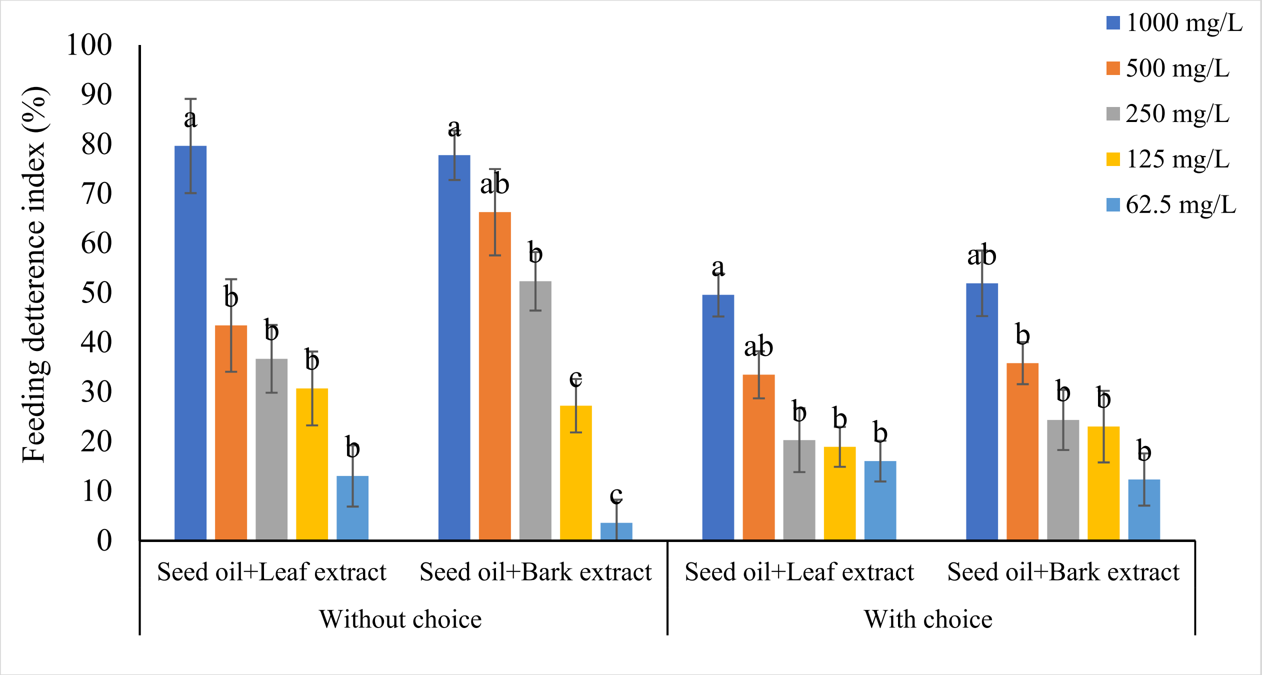


**Figure S2**. Feeding deterrence index of binary mixtures of seed oil with leaf and bark ethanol aqueous extract of *Triadica sebifera* against *Plutella xylostella* with choice and without choice; The same letters in the error bars (Mean ± SE) within the figure are not statistically different by Tukey's HSD ($P \leq 0.05$)

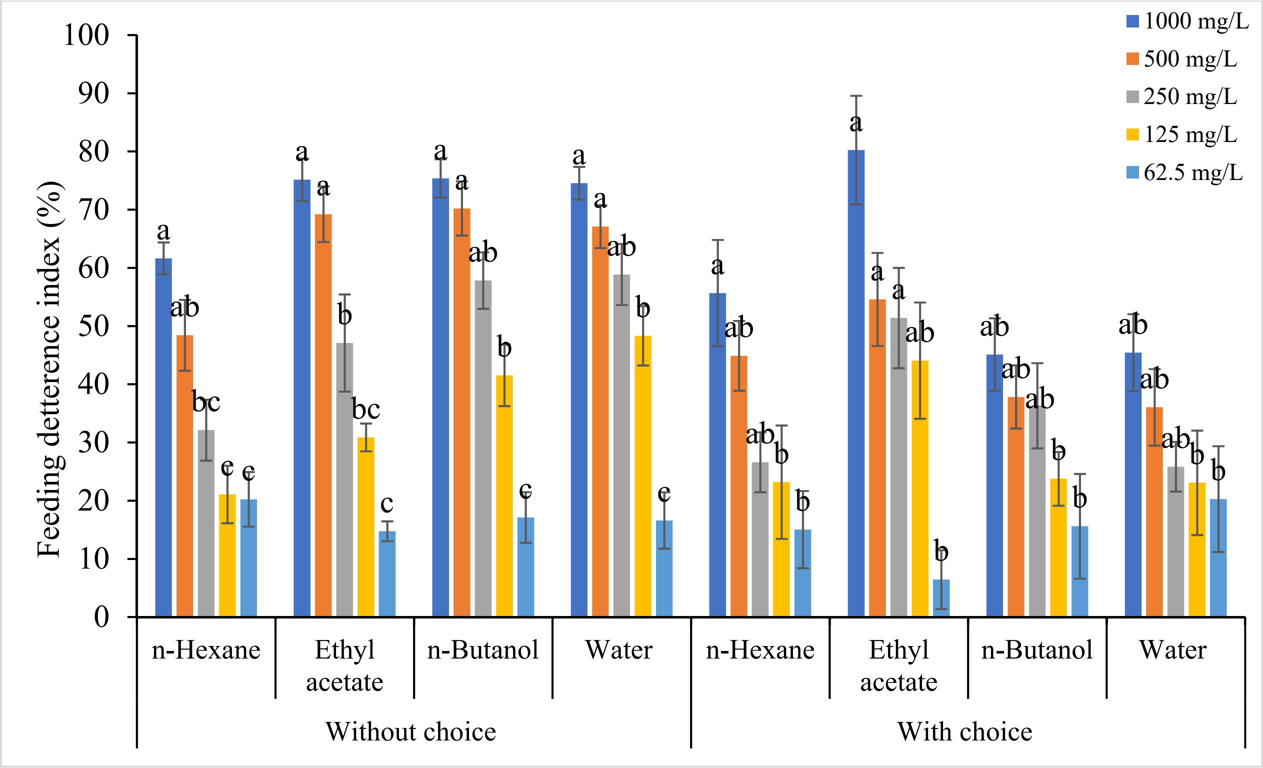


**Figure S3.** Feeding deterrence index of leaf fractions of *Triadica sebifera* against *Plutella xylostella* with choice and without choice; The same letters in the error bars (Mean ± SE) within the figure are not statistically different by Tukey's HSD ($P \leq 0.05$)

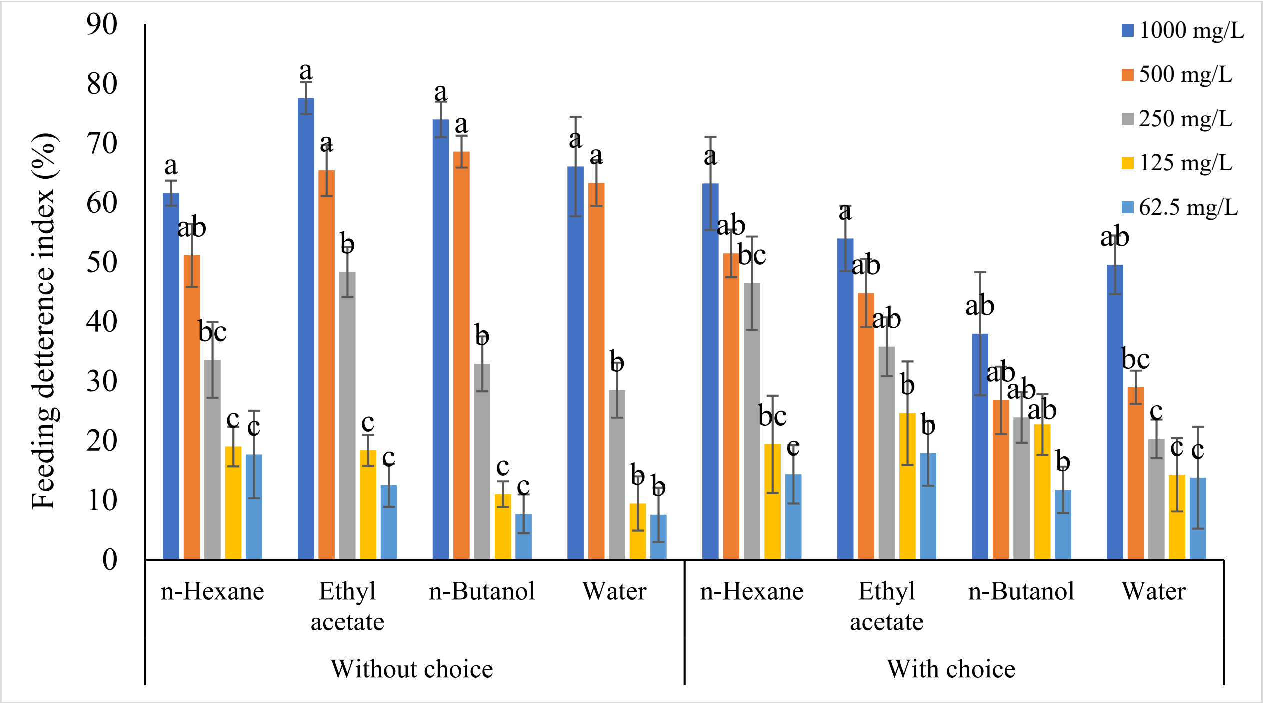


**Figure S4.** Feeding deterrence index of bark fractions of *Triadica sebifera* against *Plutella xylostella* with choice and without choice; The same letters in the error bars (Mean ± SE) within the figure are not statistically different by Tukey's HSD ($P \leq 0.05$)

**Tables S1.** Growth inhibition of leaf, bark extracts, fractions and seed oil of *Triadica sebifera* against *Plutella xylostella*

| **Extracts/oil** | $IC_{50}$ (mg/L) | Confidence limits (mg/L) | Slope ± SE | Chi square | P value |
|---|---|---|---|---|---|
| Leaf extract | 2696.49 | 2308.83 – 3161.44 | 1.82 ± 0.16 | 4.26 | 0.23 |
| Bark extract | – | – | – | – | – |
| Seed oil | 3225.25 | 2756.90 – 3811.73 | 1.79 ± 0.16 | 0.34 | 0.95 |
| **Leaf fractions** | | | | | |
| *n*-Butanol | 273.55 | 237.31 – 316.28 | 2.03 ± 0.17 | 3.40 | 0.26 |
| Water | 424.55 | 349.74 – 533.25 | 1.45 ± 0.15 | 3.45 | 0.33 |
| *n*-Hexane | – | – | – | – | – |
| Ethyl acetate | – | – | – | – | – |
| **Bark fractions** | | | | | |
| Water | 315.07 | 268.06 – 373.95 | 1.73 ± 0.16 | 0.83 | 0.84 |
| *n*-Hexane | – | – | – | – | – |
| Ethyl acetate | – | – | – | – | – |
| *n*-Butanol | – | – | – | – | – |

–No significant difference; $p<0.150$

**Tables S2.** Growth inhibition of, leaf, bark ethanol aqueous extracts, its fractions and seed oil of *Triadica sebifera* against *Plutella xylostella* (without choice method)

| Conc. (mg/L) | Growth inhibition rate (± SE) after 48 h* | | | |
|---|---|---|---|---|
| | **Leaf/bark extracts and seed oil** | | | |
| | Leaf extract | Bark extract | Seed oil | |
| 10000 | 89.40 ± 5.83 a | 99.77± 5.02 a | 82.37 ± 3.25 a | |
| 5000 | 65.86 ± 8.45 ab | 59.40 ± 6.20 b | 61.93 ± 6.10 ab | |
| 2500 | 42.34 ± 6.99 bc | 35.21 ± 9.62 bc | 41.82 ± 7.91 bc | |
| 1250 | 26.16 ± 7.98 c | 32.48 ± 5.74 bc | 22.27 ± 4.46 cd | |
| 625 | 18.07 ± 2.98 c | 7.64 ± 9.64 c | 11.04 ± 2.81 d | |
| | $F_{4,49}$=18.93; $p<0.0001$ | $F_{4,49}$=21.66; $p<0.0001$ | $F_{4,49}$=30.37; $p<0.0001$ | |
| Conc. (mg/L) | **Leaf fractions** | | | |
| | *n*-Hexane | Ethyl acetate | *n*-Butanol | Water |
| 1000 | 100.00 ± 0.00 a | 77.22 ± 4.56 a | 90.86 ± 4.88 a | 68.65 ± 5.04 a |
| 500 | 87.31 ± 5.47 a | 64.08 ± 6.20 a | 65.07 ± 9.73 ab | 56.59 ± 5.36 a |
| 250 | 50.60 ± 5.57 b | 21.44 ± 5.70 b | 44.46 ± 10.70 bc | 39.28 ± 9.37 ab |
| 125 | 38.40 ± 4.64 b | 15.91 ± 5.35 b | 29.01 ± 13.31 bc | 16.15 ± 8.79 b |
| 62.5 | 34.50 ± 6.30 b | 10.80 ± 4.86 b | 9.27 ± 9.18 c | 14.07 ± 9.35 b |
| | $F_{4,49}$=36.07; $p<0.0001$ | $F_{4,49}$=32.31; $p<0.0001$ | $F_{4,49}$=10.11; $p<0.0001$ | $F_{4,49}$=9.51; $p<0.0001$ |
| Conc. (mg/L) | **Bark fractions** | | | |
| | *n*-Hexane | Ethyl acetate | *n*-Butanol | Water |
| 1000 | 96.24 ± 2.10 a | 99.68 ± 3.03 a | 87.36 ± 7.17 a | 79.92 ± 2.95 a |
| 500 | 86.36 ± 5.29 a | 52.30 ± 8.55 b | 54.53 ± 9.57 ab | 66.21 ± 5.31 a |
| 250 | 54.36 ± 6.36 b | 46.47 ± 6.06 bc | 27.38 ± 10.76 bc | 39.89 ± 7.14 b |
| 125 | 34.58 ± 3.32 bc | 33.51 ± 8.66 bc | 21.25 ± 5.89 bc | 26.32 ± 7.96 bc |
| 62.5 | 28.57 ± 7.36 c | 20.97 ± 3.61 c | 10.05 ± 10.14 c | 10.75 ± 4.79 c |
| | $F_{4,49}$=33.26; $p<0.0001$ | $F_{4,49}$=21.73; $p<0.0001$ | $F_{4,49}$=12.18; $p<0.0001$ | $F_{4,49}$=23.06; $p<0.0001$ |

*Mean of three replications; Mean followed by the same letter within a column are not statistically different by Tukey's HSD ($P \geq 0.05$)

**Table S3.** Growth inhibition of binary mixtures of *Triadica sebifera* against *Plutella xylostella* (without choice)

| Conc. (mg/L) | Growth inhibition rate (± SE) after 48 h* | |
|---|---|---|
| | Seed oil + Leaf extract | Seed oil + Bark extract |
| 1000 | 99.87 ± 4.48 a | 98.77 ± 7.92 a |
| 500 | 88.06 ± 5.52 ab | 85.76 ± 7.63 ab |
| 250 | 80.44 ± 8.13 abc | 84.23 ± 6.81 ab |
| 125 | 63.19 ± 4.68 bc | 72.56 ± 4.62 ab |
| 62.5 | 55.15 ± 10.66 c | 54.28 ± 11.40 b |
| $F_{4,49}$ | 6.56; $p$<0.0001 | 4.34; $p$<0.005 |

*Mean of three replications; Mean followed by the same letter with in a column are not statistically different by Tukey's HSD ($p \geq 0.05$)

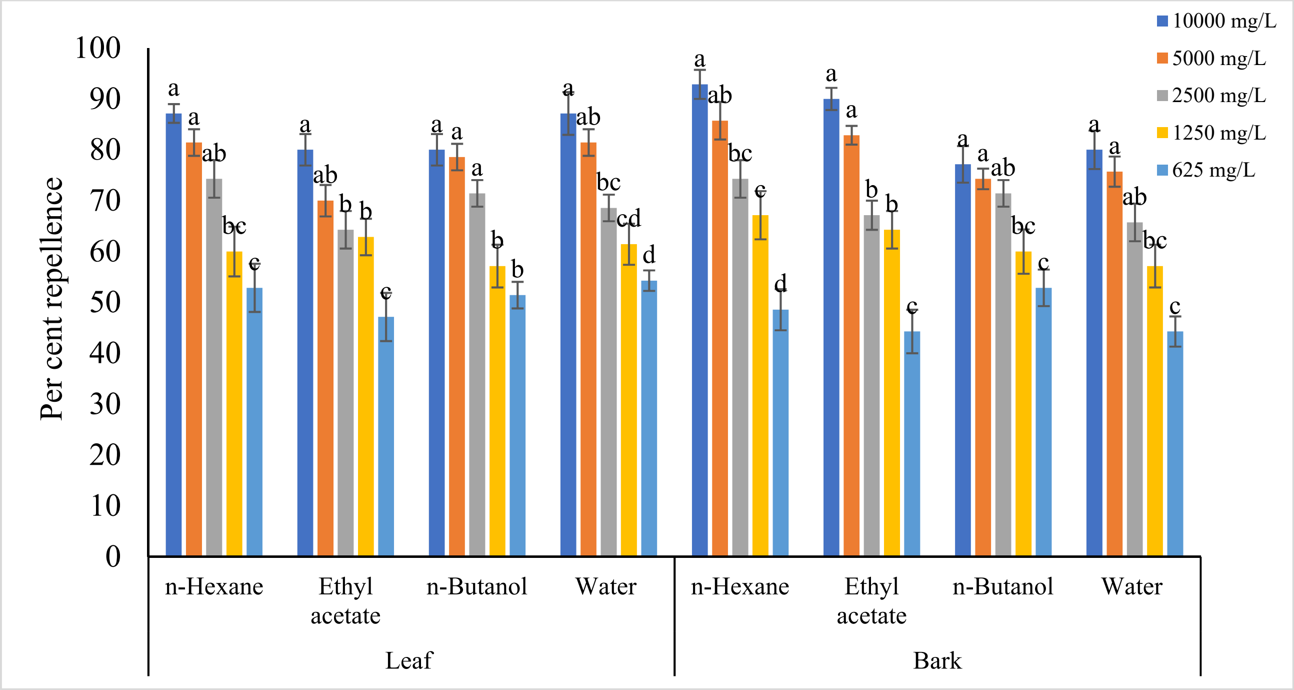


**Figure S5.** Per cent repellence of leaf and bark fractions of *Triadica sebifera* against *Plutella xylostella*; The same letters in the error bars (Mean ± SE) within the figure are not statistically different by Tukey's HSD ($P \leq 0.05$)
